# Prevalence of vulvovaginal candidiasis among pregnant women in the Ho municipality, Ghana: species identification and antifungal susceptibility of Candida isolates

**DOI:** 10.1186/s12884-020-02963-3

**Published:** 2020-05-06

**Authors:** Sayanika Devi Waikhom, Innocent Afeke, Grace Sefakor Kwawu, Hintermann Kobina Mbroh, George Yiadom Osei, Bengyella Louis, John Gameli Deku, Emmanuel Senyo Kasu, Prosper Mensah, Charles Yao Agede, Cornelius Dodoo, Emmanuel Akomanin Asiamah, John Tampuori, John Korbuvi, Japheth Awuletey Opintan

**Affiliations:** 1 Department of Biomedical Sciences, School of Basic and Biomedical Sciences, University of Health and Allied Sciences, PMB 31 Ho, Ghana; 2 Department of Medical Laboratory Sciences, School of Allied Health Sciences, University of Health and Allied Sciences, Ho, Ghana; 3 Gynecology Department, Ho Teaching Hospital, Ho, Ghana; 4 Public Health Department, Ho Teaching Hospital, Ho, Ghana; 5 Department of Biostatistics and Epidemiology, School of Public Health, University of Health and Allied Sciences, Ho, Ghana; 6 Medical Laboratory Department, Ho Teaching Hospital, Ho, Ghana; 7 Department of Pharmaceutical Microbiology, School of Pharmacy, University of Health and Allied Sciences, Ho, Ghana; 8 Urology Department, Ho Teaching Hospital, Ho, Ghana; 9 Pharmacy Department, Ho Teaching Hospital, Ho, Ghana; 10 Microbiology Department, University of Ghana Medical School, Accra, Ghana

**Keywords:** Vulvovaginal candidiasis, Symptomatic vulvovaginal candidiasis, Asymptomatic vulvovaginal candidiasis, Germ tube test, HiCrome *Candida* differential agar, Fluconazole, Voriconazole, Nystatin, Non-*albicans Candida*, *Candida albicans*

## Abstract

**Background:**

*Candida* is the leading cause of vaginitis, and 75% of women have at least one episode of infection in their lives, with pregnancy being a predisposing factor. If left untreated, vulvovaginal candidiasis (VVC) can lead to chorioamnionitis with subsequent abortion, prematurity and congenital infection of the neonate. We aimed to determine the prevalence of VVC, identify the recent and most frequently occurring species of *Candida* in pregnant women, and determine the most effective antifungal drug of choice for treatment.

**Method:**

A prospective cross-sectional study in which 176 high vaginal swab samples of consented pregnant women visiting the antenatal clinic from February 2018 to April 2018 were subjected to direct gram smear and culture for *Candida* isolation. *Candida* isolates were identified using a germ tube test and HiCrome *Candida* differential agar. *Candida* isolates were then subjected to a disk diffusion method using fluconazole (25 μg), nystatin (100 units), and voriconazole (1 μg) on Mueller-Hinton agar supplemented with 2% (w/v) glucose and 0.5 μg/ml methylene blue dye to determine the susceptibility pattern as per the guidelines of the Clinical Laboratory Standard Institute (CLSI). Chi-square analysis was used to ascertain the significant association of participants’ sociodemographics and clinical presentations to VVC. A univariate logistic regression model was used to identify potential risk factors of VVC.

**Results:**

The prevalence of VVC among our study participants was 30.7%. Non-*albicans Candida* (NAC) and *Candida albicans* had a prevalence of 74.1 and 25.9%, respectively. *Candida glabrata* was the most common species, followed by *Candida albicans*, *Candida krusei*, *and Candida parapsilosis*. 50.0, 18.5 and 3.7% of *Candida* species were susceptible to voriconazole, fluconazole and nystatin, respectively, whereas 37.0, 48.1 and 9.3% of *Candida* species were resistant to voriconazole, fluconazole and nystatin, respectively. The majority of isolates were susceptible dose dependent to all three antifungal agents, with voriconazole being the most efficacious antifungal agent. There was no significant association between participants’ socio-demographic information and clinical presentations to VVC.

**Conclusion:**

The prevalence of VVC was high in the study area. *C. glabrata* was found to be the most common cause of VVC among the pregnant women attending antenatal clinics, in the Ho Municipality region of Ghana. The majority of the *Candida* isolates were susceptible and resistant to voriconazole and fluconazole, respectively.

## Background

*Candida* is the leading cause of vaginitis, and 75% of women have at least one episode in their lifetimes [1, 2]. Pregnancy is a predisposing factor for vulvovaginal candidiasis (VVC) [3–5]. During pregnancy, there is an increase in progesterone and oestrogen levels, especially in the last trimester [6–8]. Progesterone has an inhibitory effect on the anti-*Candida* activity of neutrophils [6]. On the other hand, oestrogen reduces the ability of vaginal epithelial cells to inhibit the growth of *Candida albicans* on them [9]. However, about 75% of women generally harbour this fungus without it causing harm to them [10–12]. During normal pregnancy, candidiasis is frequently encountered without significant risk for the foetus. Nevertheless, pregnancy may be negatively affected by VVC. If untreated, vaginal candidiasis can lead to chorioamnionitis with subsequent abortion and prematurity in pregnant women, congenital infection of the neonate and pelvic inflammatory disease (PID) resulting in infertility in non-pregnant women [12]. VVC could be a risk factor for candidemia in preterm neonates during normal pregnancy. Amongst the *Candida* species isolated from vaginal specimens, *C. albicans* is the most predominant, followed by other non-*albicans Candida* (NAC) such as *C. glabrata, C. tropicalis, C. dubliniensis* and *C. krusei* [13–15]. With the increase in frequency of non-*albicans Candida* being isolated from clinical specimens, recent studies indicated that non-*albicans Candida* are now considered pathogens [16, 17]. As a result, compared to *Candida albicans*, NAC are developing resistance to most antifungals used as therapy to treat VVC [16, 17]. This is widely attributed to the use of over the counter (OTC) drugs and empiric regimes to treat these infections, since speciation and antifungal susceptibility testing of *Candida* isolates are not done routinely for clinical purposes [18, 19] in Ghana. Most frequently, azole-based drugs are the drug of choice for treating *Candida* infections [20]. In Ghana, there is paucity of data regarding the prevalence of VVC, its distribution and the in vitro antifungal susceptibility pattern of *Candida* isolates from vaginal swabs of pregnant women. With the increasing shift in population of *Candida* species, from *C. albicans* to NAC, and the rapid development of resistance to drugs being administered, it is imperative to study the antifungal susceptibility of *Candida* species among pregnant women in the Ho Municipality. Early diagnosis and appropriate treatment may improve the pregnant women’s and neonates’ clinical conditions [21]. Identification of *Candida* species in human infections has increased, and may partly be due to improvements in diagnostic methods such as the use of chromogenic media, which has the ability to differentiate pathogenic *Candida* isolates into their individual species [22]. Chromogenic agar is rapid, simple and cost effective compared to conventional methods, which are slow, technically demanding and expensive [23, 24].

This study was designed to ascertain the prevalence of VVC, identify the recent and most occurring species of *Candida* associated with VVC in pregnant women and determine the most effective antifungal drug for treatment.

## Methods

### Study site and study design

This prospective cross-sectional study enrolled 176 pregnant women with complete data who visited the antenatal clinics (ANC) at the Ho Teaching Hospital (HTH) and the Ho Municipal Hospital (HMH) from February 2018 to April 2018. HTH is the major referral center in the Volta Region. These health facilities are situated in Ho, the capital city of the Volta Region. At average, 20 and 15 pregnant women are seen at the ANC on a daily basis at the HTH and HMH, respectively. Processing of samples was done at the Microbiology unit of the HTH laboratory.

### Study population, participant’s selection and recruitment

Pregnant women in any trimester who visited the ANC were interviewed one-on-one and those that gave consent to be part of the study were enrolled. Pregnant women who had any pregnancy related complications (diabetes, bleeding per vagina, hypertension) or were on either antifungal agents/antibiotics were excluded in the study. Pregnant women were enrolled once to avoid repetition.

### Study procedure

Information regarding participants’ socio-demographics (age, educational level, marital status, religion, trimester in pregnancy) and clinical presentations (burning sensation, discharge and irritation) were extracted from participants’ ANC books. Information that were not readily available in the ANC books were collected using semi-structured questionnaire administered to participants of the study. Patients were assigned study identification numbers (IDs) which were used throughout the study. The IDs were generated by using the initials of the hospital where participants were enrolled and a chronological number according to how the patients were recruited.

After detailed explanation of the sampling procedure to the participants, two high vaginal swabs (HVS) were obtained by a Gynecologist/trained midwife from each participant using sterile swab sticks. The samples were labelled using generated IDs of study participants. Each participant was made to lie in a lithotomic position where the vaginal walls were examined for white patches. Using a speculum, the labia of the vagina was opened, and a sterile swab stick was inserted into the vagina to the posterior fornix where the swab stick was gently rotated around to soak fluid unto the sterile cotton bud. The procedure was repeated to obtain two HVS. Samples obtained were transported in Amies transport medium to the laboratory within 1 h for bacteriological analysis. Based on the combination of the clinical presentations, Gram and culture outcome, participants were categorized into three groups; symptomatic, asymptomatic and negative VVC. Symptomatic vulvovaginal candidiasis was defined as the presence of clinical presentations (burning sensation, discharge, and irritation) and the presence of yeast-liked cells in direct Gram smear with positive culture. Asymptomatic VVC (colonization) was defined as the absence of clinical presentations (burning sensation, discharge, and irritation) but the presence of yeast-liked cells in direct Gram smear with positive or negative culture. A negative case was defined as the absence of clinical presentations and the absence of yeast-like cells in direct Gram smear with negative culture.

### Laboratory test processes

**Fig. 1:**
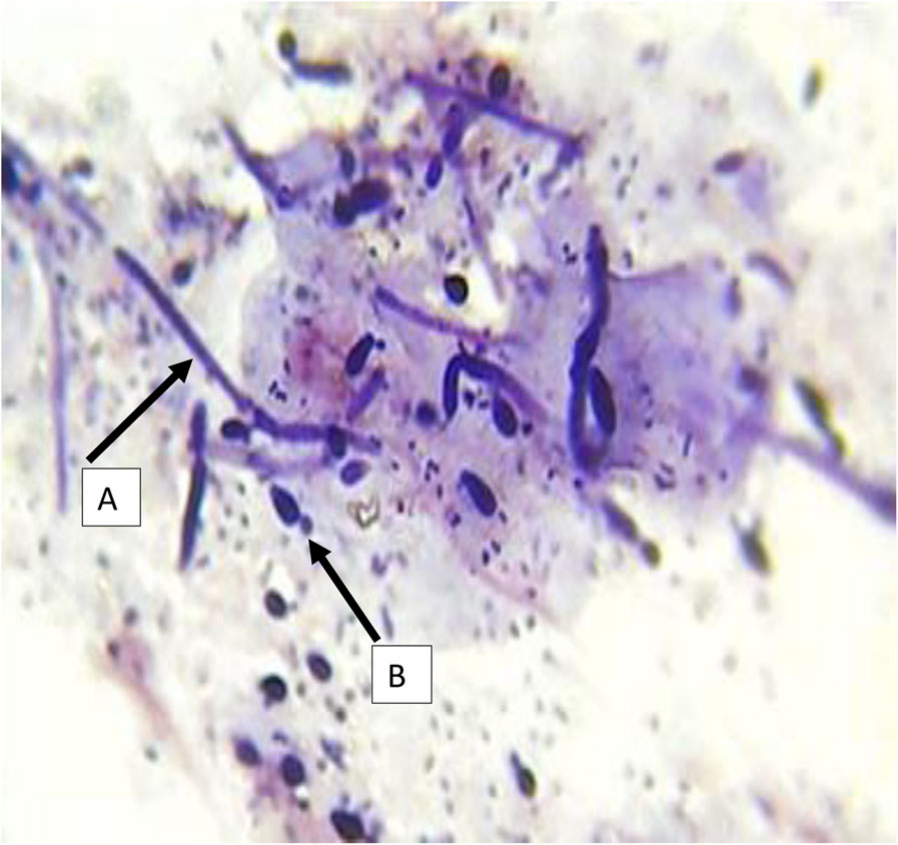
**a**-Micrograph of Pseudohyphae of *Candida albicans* in direct Gram smear of HVS; **b**-Micrograph of Blastoconidia (budding yeast-like cell) of *Candida albicans* in direct Gram smear of HVS (×100)

HVS samples were processed at the Microbiology laboratory of the Ho Teaching Hospital within 1 h of collection. One swab was used for culture and the other for direct Gram smear for Gram positive oval-shaped organisms with buds and/or pseudohyphae/hyphae (Fig. 1).

**Fig. 2:**
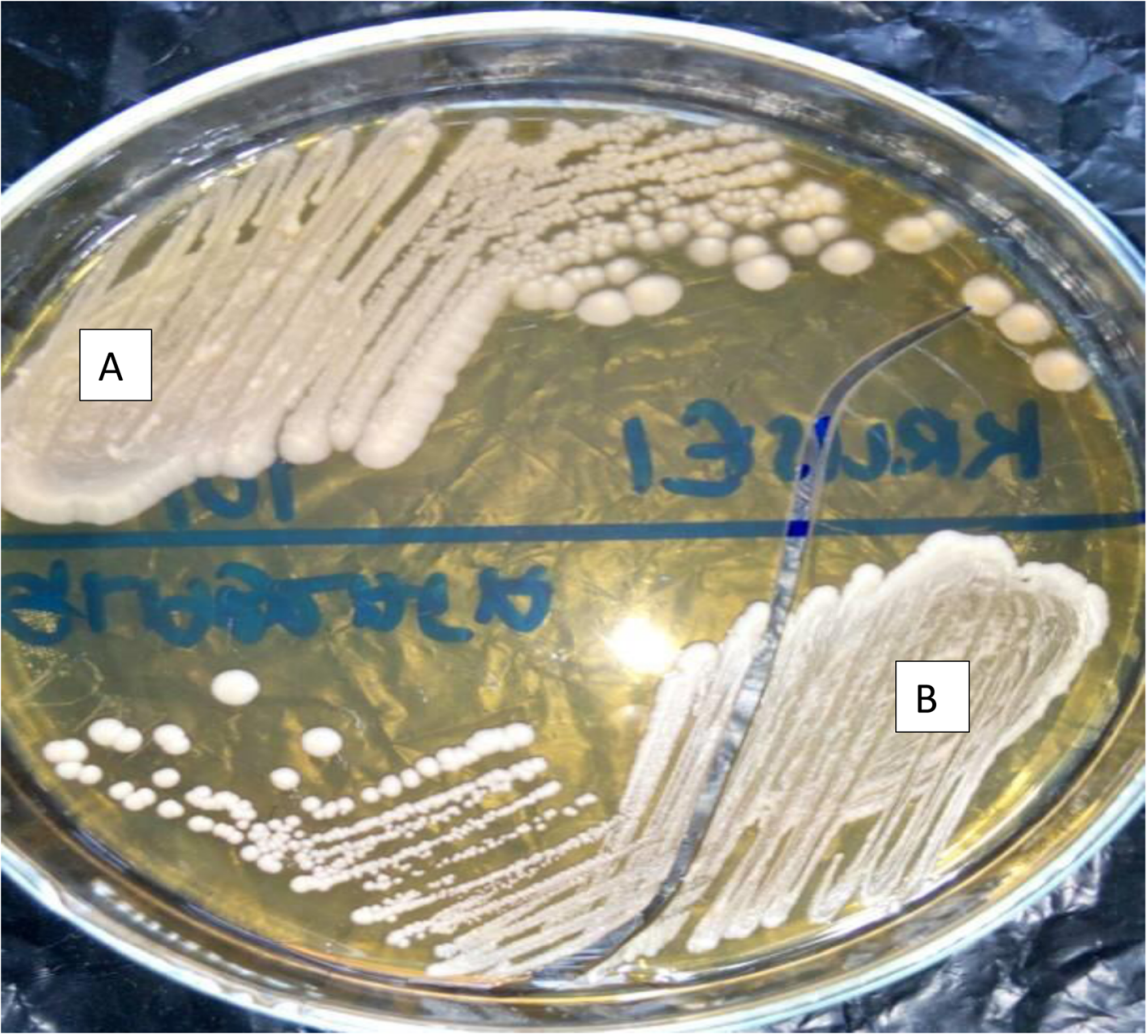
**a**-Photograph of *Candida krusei* (cream, dry, dull, convex colonies) on Sabouraud Dextrose Agar; **b***-*Photograph of *Candida albicans* (pale cream, smooth, glistening, convex colonies) on Sabouraud Dextrose Agar after 48 h of aerobic incubation at 37 °C

Swabs were inoculated on Sabouraud Dextrose agar (SDA) (TPC, India) plates and incubated aerobically at 37 °C, for 24 h. Plates with no growth after 24 h were re-incubated for another 24 h [25, 26] (Fig. 2). Colonies on SDA plates were subjected to colony identification and Gram staining for yeast-like cells. All isolates (54) were stored in Brain Heart Infusion (BHI) (Oxoid Ltd., England) with 15% glycerol at − 20 °C for further research.

**Fig. 3:**
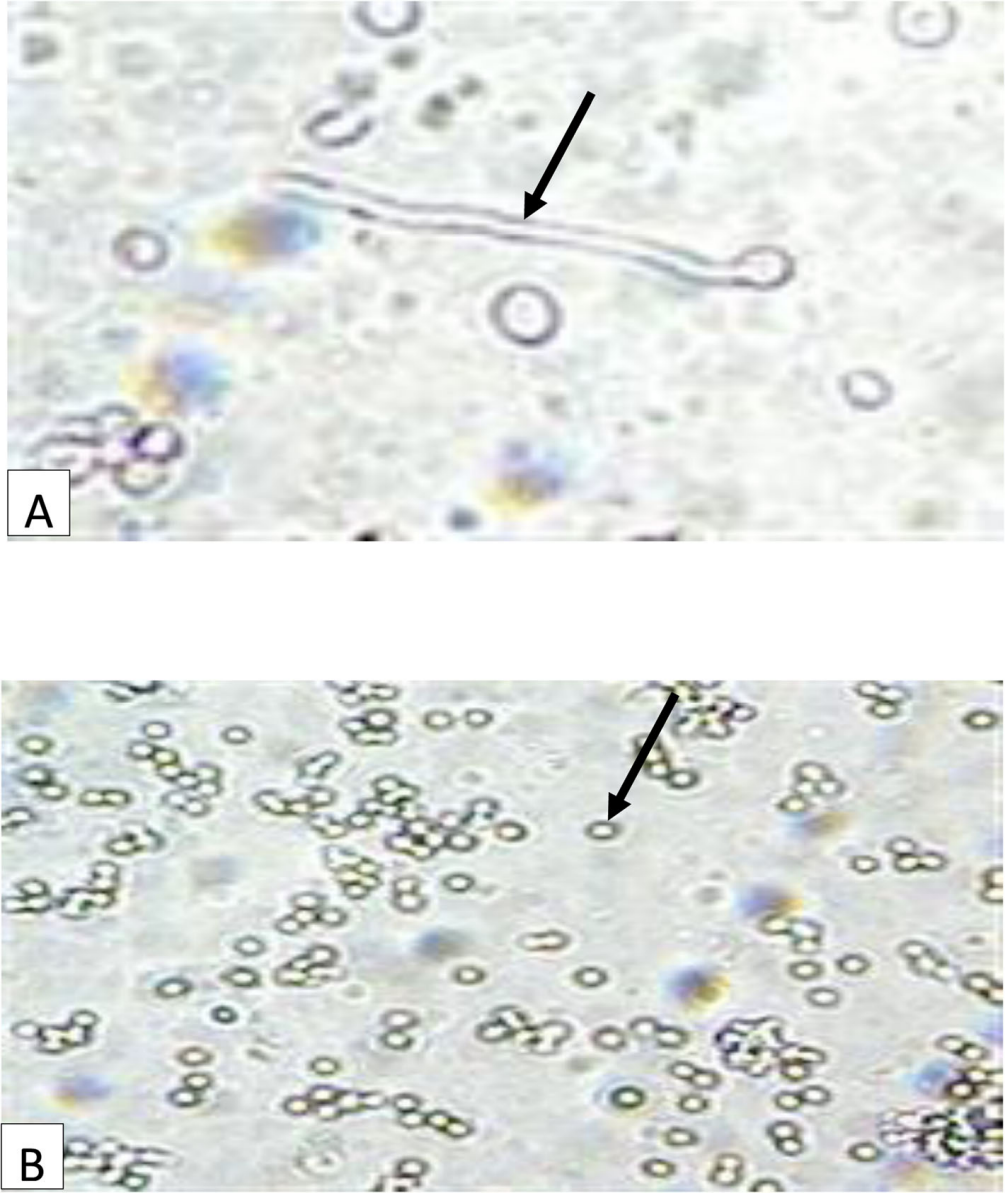
Micrograph of Germ tube formation in *Candida* species**; a**- *C. albicans* (short, slender tube structure without constrictions- Germ tube positive); **b**- *C. glabrata* (oval budding yeast cells without pseudohyphae- Germ tube negative) after 4 h of aerobic incubation (×40)

Yeast-like cells were speciated by subjecting them to germ tube test. Small portions of isolated colonies of the yeasts were suspended in test tubes containing 0.5 ml human serum. The test tubes were incubated at 35 °C for 4 h. A drop of the well-mixed incubated suspensions was placed on clean grease-free microscope slides with a cover slip and observed using × 40 objective magnification. Filamentous extension from yeast cell with no constriction at the neck was considered as germ tube positive. Isolates with no extensions or extensions with constrictions at the neck were considered germ tube negative [27] (Fig. 3).

**Fig. 4:**
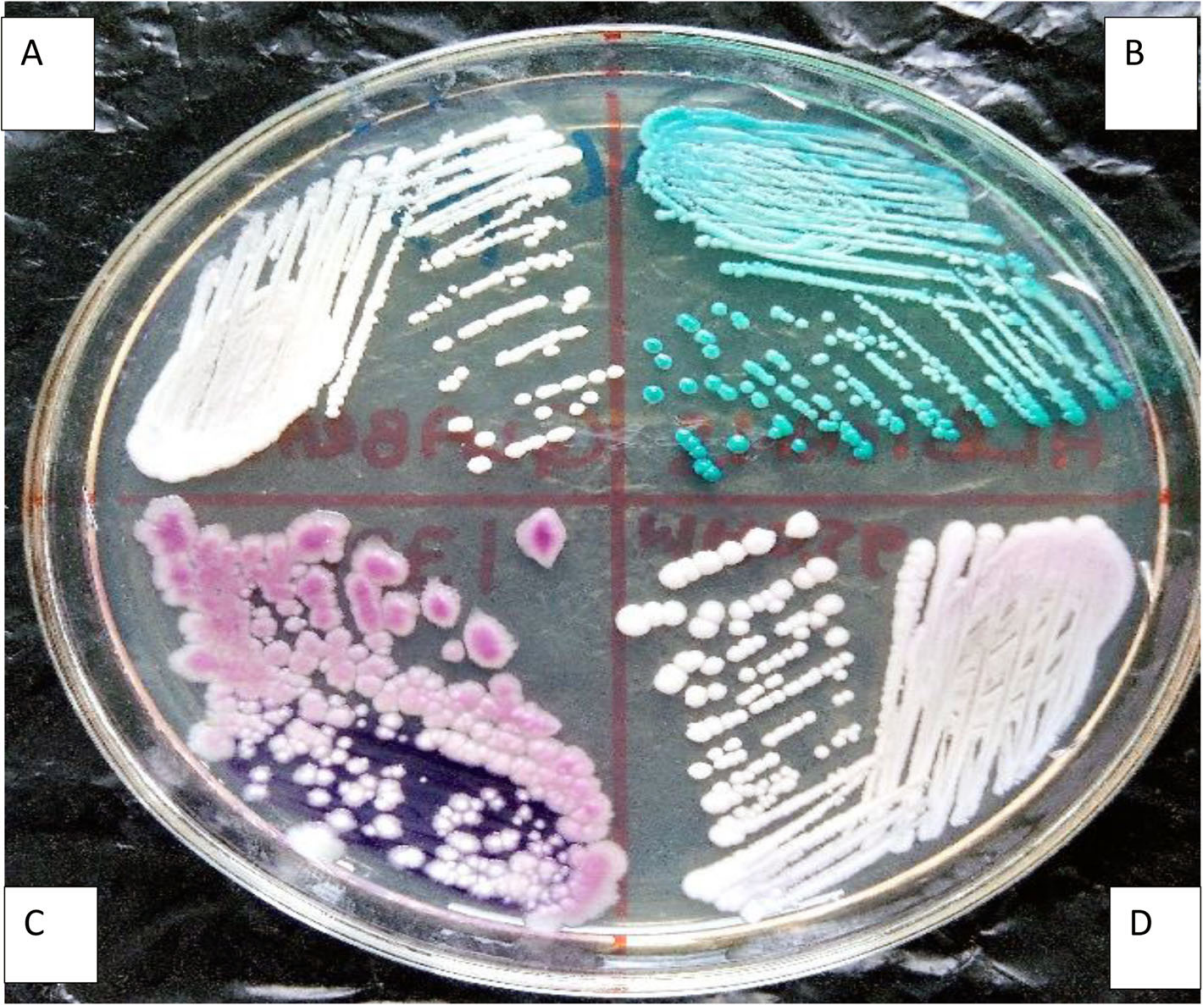
Photograph of *Candida* species as seen on HiCrome agar **a**: Cream coloured, glistening, smooth, convex colonies of *C. glabrata* on HiCrome *Candida* Differential agar **b**: Light green coloured, glistening, smooth, convex colonies of *C. albicans* on *Candida* Differential agar **C**: Purple coloured, fuzzy, dull, flat, irregular colonies of *C. krusei* on HiCrome *Candida* Differential agar **D**: Cream to pale pink coloured, glistening, smooth, convex colonies of *C. parapsilosis* on HiCrome *Candida* Differential agar after 48 h of aerobic incubation at 37 °C

Isolates were sub-cultured on HiCrome *Candida* differential agar (HiMedia, India) and incubated aerobically for 48 h [28]. Colonies on HiCrome *Candida* differential agar were identified by colour, appearance and shape (Table 1; Fig. 4).

**Table 1:** Differentiation of *Candida* with HiCrome *Candida* Differential Agar [29, 30]

Species	Description on HiCrome ***Candida*** Differential Agar
*C. glabrata*	Cream, glistening, convex, smooth
*C. krusei*	Purple, fuzzy, dull, flat, irregular
*C. parapsilosis*	Cream to pale pink, glistening, smooth, slightly raised
*C. albicans*	Light green, glistening, smooth, convex

**Fig. 5:**
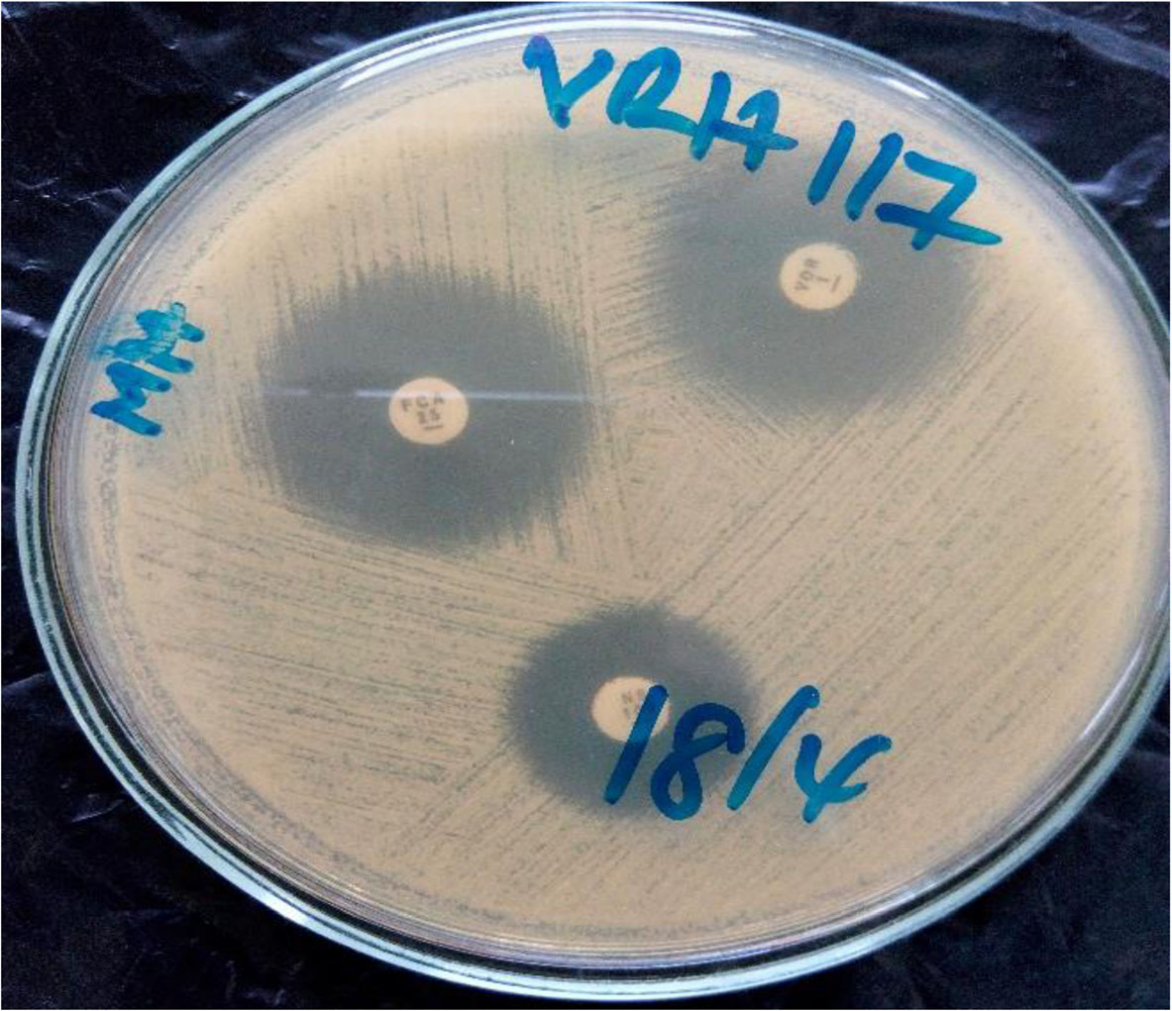
Photograph of Antifungal susceptibility testing of Fluconazole (25 μg), Nystatin (100 units), and Voriconazole (1 μg) showing zone of inhibition on Muellar Hinton agar supplemented with 2% glucose and 0.5 μl of methylene blue after 24 h of aerobic incubation at 37 °C

Antifungal susceptibility testing was performed on Mueller-Hinton (MH) agar (Oxoid Ltd., England) supplemented with 2% glucose and 0.5 μg/ml methylene blue by disk diffusion method for nystatin (100 μg), fluconazole (25 μg) and voriconazole (1 μg) disks (Oxoid Ltd., England) [31, 32]. Using a sterile inoculating loop, an inoculum was prepared using distinct colonies of *Candida* isolates from the SDA plates which were transferred into a 5 ml test tube containing 0.85% sterile saline solution, and emulsified to form a suspension of turbidity equivalent to 0.5 McFarland standard as compared to a 0.5 McF PhoenixSpec Calibrator (Becton, Dickinson and Company, USA). The lawn of the media was seeded in three dimensions using sterile swabs dipped in prepared inoculum. Antifungal disks were then placed aseptically on the lawn and incubated at 37 °C for 24 h. Plates that did not register growth were re-incubated for extra 24 h [32] (Fig. 5). Zone diameters of antifungal disks were read using measuring ruler. For azoles (fluconazole, voriconazole), zones were read up to colonies of normal size; and zones of growth of partially inhibited colonies whose sizes were smaller nearer the disk than at the edge of the real zone which were not seen was considered as resistant mutants. For nystatin, clear zones with no visible growth were read. If colonies were seen inside the zone, they were considered resistant mutants [32] (Table 2). Quality control was ensured using *Candida albicans* ATCC 44374. Fifty percent of all setups were randomly selected and were re-examined by two other readers to ensure quality.

**Table 2:** Zone diameter Interpretive Standards [31, 33]

Antifungal agents	Zone diameter in mm		
	Resistant (mm)	Susceptible Dose Dependent (mm)	Susceptible (mm)
Fluconazole (25 μg)	≤14	15–18	≥19
Voriconazole (1 μg)	≤13	14–16	≥17
Nystatin (100 units)	≤16	17–24	≥25

### Sample size estimation

A sample size of 255 was obtained per calculation based on a previous data which showed that 21% of VVC is prevalent among pregnant women in Ghana [13] and a 95% confidence level with a 5% allowable error.

### Data collection, management and analysis

Both clinical and laboratory data were extracted and recorded manually into an MS-office Excel software Version 1908 (Microsoft Corporation Copyright 2016). Data was later analyzed using Statistical Package for the Social Sciences Version 22 (SPSS, SPSS Inc., Chicago, IL, USA). Categorical data variables were statistically described in the form of frequencies and percentages while continuous data variables were summarized as mean (Standard Deviation). The association between categorical variables were done using chi-square test. *P*-value was considered significant if less than 0.05. Univariate logistic regression model was used to identify potential risk factors of VVC. The association between VVC and clinical presentations of study participants was determined using chi-square test.

## Results

### Characteristics of study participants

**Fig. 6:**
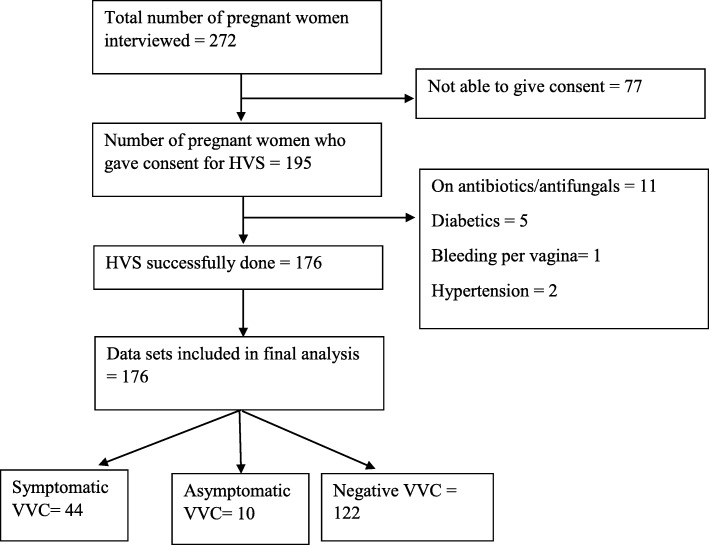
Study participant flow

A total of 272 participants were interviewed, but only 176 of them were eligible and consented to be part of the study. The remaining 96 pregnant women were either not eligible or verbally declined to be part of the study (Fig. 6).

Complete socio-demographic data, clinical presentations and vaginal swabs were taken from all 176 consenting participants. Participants’ mean age was 29 years, with a range of 15–42 years. Most (59.7%) of the participants were in their third trimester. The majority, 73.9% were married, with 51.7% having acquired basic education. Only 2.8% had no formal education. A large majority (96.6%) were Christians (Table 3).

**Table 3:** General demographic information of study participants (*N* = 176)

Parameter	Frequency		Percentage (%)
Total respondents	176		100
Age (in years)	29 (6)	Range (15–42)	
**Age group (in years)**			
< 20	12		6.8
20–29	85		48.3
30–39	74		42.0
40–49	5		2.8
**Educational Status**			
None	6		3.4
Basic	91		51.7
Secondary	35		19.9
Tertiary	44		25.0
**Marital Status**			
Single	33		18.8
Co-habiting	13		7.4
Married	130		73.9
**Religion**			
Muslim	6		3.4
Christian	170		96.6
**Trimester in pregnancy**			
First	28		15.9
Second	43		24.4
Third	105		59.7
**Candida Infection**			
Negative	122		69.3
Positive (VVC)	54		30.7
➣ Symptomatic VVC	44		81.5
• *C. albicans*	12		27.3
• *C. parapsilosis*	1		2.3
• *C. glabrata*	25		56.8
• *C. krusei*	6		13.6
➣ Asymptomatic VVC	10		18.5
• *C. albicans*	2		20.0
• *C. parapsilosis*	2		20.0
• *C. glabrata*	6		60.0
• *C. krusei*	0		0.0

Data is presented as frequency and percentage

Mean age ± Standard Deviation

*VVC* Vulvovaginal Candidiasis

Out of the 176 pregnant women, 4.5% had all three clinical presentations, with one of them diagnosed as having VVC. Approximately, 33.5%, had discharge; the majority of these, 33.3%, were VVC-confirmed participants. There was no significant association between symptoms and VVC in general, except between burning sensation and irritation (Table 4).

**Table 4:** Distribution of signs and symptoms among study participants

SYMPTOMS	TOTAL	Positive	OR (95% CI)	*P*-value
All Symptoms	8 (4.5)	1 (1.9)	0.50 (0.06–4.56)	0.539
Burning Sensation & Discharge only	3 (1.7)	2 (3.7)	7.00 (0.57–85.38)	0.127
Burning Sensation & Irritation only	11 (6.3)	7 (13.0)	6.13 (1.49–25.22)	0.012*
Burning Sensation only	4 (2.3)	1 (1.9)	1.17 (0.11–12.48)	0.899
Discharge only	59 (33.5)	18 (33.3)	1.54 (0.63–3.76)	0.347
Irritation & Discharge only	37 (21.0)	13 (24.1)	1.90 (0.72–5.02)	0.198
Irritation only	9 (5.1)	2 (3.7)	1.02 (0.18–5.59)	0.988
No symptom	45 (25.6)	10 (18.5)	1	
Total	**176 (100.0)**	**54 (100.0)**		

X^2^ = 0.143

Data is presented as frequency and percentage in parenthesis

Positive represents pregnant women who were culture positive for VVC

X^2^ is the *p*-value obtained with the chi-square test

Among the 54 VVC-confirmed study participants, the highest frequency of *Candida* isolates was recorded in the age range of 20 to 29 years, with the lowest frequency in the range of 40 to 49 years. Over half, or 53.7%, had basic education. A majority, or 66.7%, of the VVC participants were married, with the lowest frequencies recorded in co-habiting participants. There was no statistically significant association between participants’ socio-demographics and the frequency of VVC (*P* > 0.05) (Table 5).

**Table 5:** Association between socio-demographic characteristics and vulvovaginal candidiasis (*N* = 176)

	VVC		OR (Unadjusted)	*P*-value for OR	AOR (Adjusted)	*P*-value for AOR
	Positive n (%)	Negative n (%)				
Total respondents	54 (30.7)	122 (69.3)				
**Age group (in years)**						
< 20	6 (11.1)	6 (4.9)	1.0	–	–	–
20–29	24 (44.4)	61 (50.0)	0.67 (0.20–2.23)	0.514	–	–
30–39	22 (40.7)	52 (42.6)	0.88 (0.24–3.19)	0.848	–	–
40–49	2 (3.7)	3 (2.5)	0.88 (0.62–12.45)	0.925	–	–
**Educational Status**						
None	1 (1.9)	5 (4.1)	1.0	–	–	–
Basic	29 (53.7)	62 (50.8)	0.77 (0.07–9.15)	0.837	–	–
Secondary	12 (22.2)	23 (18.9)	0.80 (0.06–10.29)	0.867	–	–
Tertiary	12 (22.2)	32 (26.2)	0.62 (0.05–7.76)	0.711	–	–
**Marital Status**						
Single	12 (22.2)	21 (17.2)	1.0	–	–	–
Co-habitation	6 (11.1)	7 (5.7)	0.73 (0.31–1.72)	0.469	–	–
Married	36 (66.7)	94 (77.0)	1.47 (0.38–5.62)	0.575	–	–
**Religion**						
Muslim	0 (0.0)	6 (4.9)	1.0	–	–	–
Christian	54 (100.0)	116 (95.1)	0.0	–	–	–
**Trimester in pregnancy**						
First	9 (16.7)	19 (15.6)	1.0	–	–	–
Second	14 (25.9)	29 (23.8)	0.98 (0.34–2.81)	0.963	–	–
Third	31 (57.4)	74 (60.7)	0.86 (0.34–2.19)	0.755	–	–

Data is presented as frequency and percentage in parenthesis

### Prevalence of VVC

The prevalence of VVC among our study participants was 30.7%. Participants in their third trimester contributed to 57.4% of the VVC prevalence. Statistically, there was no significant association between symptoms and trimester of pregnancy (Table 6). Out of the 54 VVC confirmed participants, 81.5% had symptomatic VVC, while 18.5% were colonized (asymptomatic). *C. glabrata* formed 57.4% of all *Candida* isolates among both colonized and symptomatic VVC participants, followed by *C. albicans*, with 25.9% prevalence (Table 3).

**Table 6:** Prevalence of symptoms of VVC in relation to trimester of pregnancy

Symptoms	1st trimester	2nd trimester	3rd trimester	Total
All Symptoms	1 (3.6)	3 (7.0)	4 (3.8)	8 (4.5)
Burning Sensation & Discharge only	1 (3.6)	1 (2.3)	1 (1.0)	3 (1.7)
Burning Sensation & Irritation only	1 (3.6)	3 (7.0)	7 (6.7)	11 (6.3)
Burning Sensation only	1 (3.6)	0	3 (2.9)	4 (2.3)
Discharge only	8 (28.6)	15 (34.9)	36 (34.3)	59 (33.5)
Irritation & Discharge only	7 (25.0)	8 (18.6)	22 (21.0)	37 (21.0)
Irritation only	1 (3.6)	2 (4.7)	6 (5.7)	9 (5.1)
No symptom	8 (28.6)	11 (25.6)	26 (24.8)	45 (25.6)
Total	28 (100.0)	43 (100.0)	105 (100.0)	176 (100.0)

X^2^ = 0.992

Data is presented as frequency and percentage in parenthesis

X^2^ is the *p*-value obtained with the chi-square test

### Species identification

Germ tube tests and cultures on HiCrome *Candida* differential agar were employed in the identification of *Candida*. Using HiCrome agar, 74.1% NAC and 25.9% *C. albicans* were identified. *C. glabrata* was the commonest isolated species, with a frequency of 57.4%. About 75.9% of the *Candida* species were germ tube negative, while 24.1% were germ tube positive. 85.7% *C. albicans* were germ tube positive, while 96.8% of *C. glabrata* were germ tube negative.

### Antifungal susceptibility outcome

**Fig. 7:**
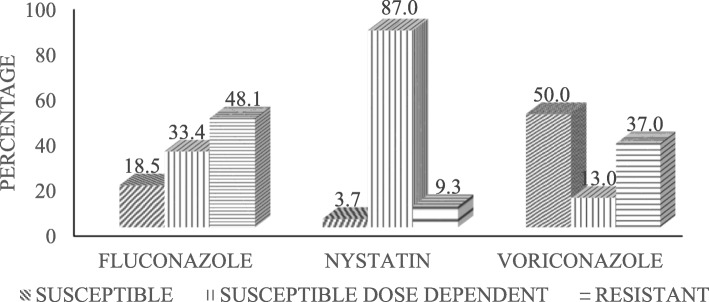
A graph showing the general In vitro antifungal susceptibility pattern of *Candida* isolates to Fluconazole (25 μg), Nystatin (100 units) and Voriconazole (1 μg) (*n* = 54)

The highest overall resistance rate of *Candida* species was seen against fluconazole (48.1%), followed by voriconazole (37.0%) and nystatin (9.3%). On the other hand, *Candida* spp. were found to be highly susceptible to voriconazole (50.0%), followed by fluconazole (18.5%) and nystatin (3.7%). Most of the *Candida* spp. were susceptible dose dependent (SDD) against nystatin (87.0%) (Fig. 7).

About 21% of *C. albicans* were susceptible and 50.0% were resistant to fluconazole. Among the NAC species, 17.5% were susceptible and 47.5% were resistant to fluconazole. When treated with nystatin, 5.0% of NAC were susceptible and 12.5% were resistant, respectively. The majority of the NAC spp. (82.5%) were SDD against nystatin. When treated with voriconazole, 42.9 and 50.0% of *C. albicans* were susceptible and resistant, respectively, while 52.5% of the NAC species were susceptible and 32.5% were resistant to voriconazole. Among the NAC species, 19.4, 41.9 and 38.7% of *C. glabrata* were susceptible, SDD and resistant to fluconazole, respectively. None of *C. glabrata* were susceptible to nystatin, but 87.1% were SDD, with a few (12.9%) being resistant. Most of *C. glabrata* (54.8%) were susceptible to voriconazole, while 35.5% were resistant. All *C. krusei* were resistant to fluconazole (Table 7).

**Table 7:** In vitro Susceptibility patterns of *C. albicans* and non-*albicans Candida* species to Fluconazole (25 μg), Nystatin (100 units) and Voriconazole (1 μg) (*n* = 54)

DRUGS	*CANDIDA* SPECIES	S n (%)	S-DD n (%)	R n (%)	Total (%)
FLUCONAZOLE	*C. albicans*	3 (21.4)	4 (28.6)	7 (50.0)	14 (100.0)
	Non*-albicans Candida*	7 (17.5)	14 (35.0)	19 (47.5)	40 (100.0)
	*C. glabrata*	6 (19.4)	13 (41.9)	12 (38.7)	31 (100.0)
	*C. krusei*	0 (0.0)	0 (0.0)	6 (100.0)	6 (100.0)
	*C. parapsilosis*	1 (33.3)	1 (33.3)	1 (33.3)	3 (100.0)
NYSTATIN	*C. albicans*	0 (0.0)	14 (100.0)	0 (0.0)	14 (100.0)
	Non*-albicans Candida*	2 (5.0)	33 (82.5)	5 (12.5)	40 (100.0)
	*C. glabrata*	0 (0.0)	27 (87.1)	4 (12.9)	31 (100.0)
	*C. krusei*	0 (0.0)	5 (83.3)	1 (16.7)	6 (100.0)
	*C. parapsilosis*	2 (66.7)	1 (33.3)	0 (0.0)	3 (100.0)
VORICONAZOLE	*C. albicans*	6 (42.9)	1 (7.1)	7 (50.0)	14 (100.0)
	Non-*albicans Candida*	21 (52.5)	6 (15.0)	13 (32.5)	40 (100.0)
	*C. glabrata*	17 (54.8)	3 (9.7)	11 (35.5)	31 (100.0)
	*C. krusei*	2 (33.3)	3 (50.0)	1 (16.7)	6 (100.0)
	*C. parapsilosis*	2 (66.7)	0 (0.0)	1(.3)	3 (100.0)

Data is presented as frequency and percentage in parenthesis

*S* Susceptible

*SDD* Susceptible Dose Dependent

*R* Resistant

## Discussion

This cross-sectional study was conducted to ascertain the prevalence of VVC, species identification and antifungal susceptibility of *Candida* isolates from pregnant women at an antenatal clinic. This study is important, since diagnosis and treatment of VVC in Ghana are mostly done on the basis of clinical presentations, without any laboratory diagnosis. The mean age of the study participants was 29 years, which correlates with the mean age of 31.5 years reported by Sasikala et al. [15]. On the contrary, a higher mean age of 37.3 years was reported by Amar et al. [34]. Most of our participants were within the range of 20–29 years, followed by 30–39 years, which correlates with the previous reports [34, 35]. However, this was not statistically significant (*p* = 0.653; *p* > 0.05). The highest frequency of VVC in pregnant women in the same age groups was reported by Rati et al. [36] and Damen et al. [17]. Although the association was not statistically significant, the majority of the VVC confirmed participants were in their third trimester [6–8] and had only basic education. Low educational background may correlate with poor personal hygiene and/or low economic status, which may, in turn, make the pregnant women prone to VVC [26]. Most of the VVC-confirmed participants in our study were married. This was in contradiction to what Bitew and Abebaw [26] reported; the majority of their VVC study participants were divorced or unmarried.

Clinical presentations reported by study subjects included burning sensation around the vulva, irritation/itching of the vulva and creamy/whitish/yellowish vaginal discharge. Although most of the study participants (35.5%) had vaginal discharge, it had no statistical association with VVC (*P* = 0.143; *P* > 0.05). Most participants had one or more of the three clinical presentations, but laboratory outcomes did not confirm them as having VVC. This outcome simply explains that clinical presentations of VVC are not pathognomonic due to the fact that other vaginal conditions may establish such presentations [2, 37]. It is therefore imperative to couple clinical presentations with laboratory outcomes in the diagnosis of VVC in pregnant women [2].

Of the 176 subjects recruited in this study, 54 were confirmed to have VVC, resulting in a prevalence rate of 30.7% among the participants. The prevalence rates we found are slightly higher than those reported by previous studies [13, 16, 17, 19]. However, our data were more or less consistent with the frequencies reported by previous studies conducted in India (36%), Uganda (45.4%), and in the middle belt of Ghana (36.5%) [15, 38, 39]. Notably, the highest frequency of VVC in our study was recorded in pregnant women in their third trimester (57.4%). A similar observation was also reported in a study conducted by Babić et al. [40]. This is because during pregnancy, mostly in the third trimester, the high levels of oestrogen result in higher glycogen deposits in the vagina, and this provides a good source of carbon, which supports the proliferation of *Candida* spp. Moreover, oestrogen increases the affinity of *Candida* to the yeast cytosol receptor in vaginal epithelial cells [40].

It is reported that about 70–85% pregnant mothers with VVC subpartally contaminate their infants with *Candida* species [41]. During chidbirth, there could be subpartal transmission of *Candida* spp. from the vagina of the mother to the neonate. This may lead to generalised fungal infection in babies, especially premature neonates, due to their immature immunity [41]. Therefore, pregnant women with VVC in their first and second trimesters have a high chance of having *Candida* chorioamnionitis, which may cause abortion [42]. VVC may also lead to low birth weights and preterm babies [43, 44]. In addition, participants with VVC may stand a risk of developing nipple candidiasis during breastfeeding when their babies develop oral thrush [45]. It is therefore imperative to offer prepartal treatment to pregnant women diagnosed with VVC [41].

We found that HiCrome *Candida* differential agar is efficient for routine identification of *Candida* isolates in the clinical setting. Our study showed that the HiCrome is effective for identification of four medically important *Candida* isolates to the species level (Table 1). It has more advantage than the germ tube test, which could only categorise isolates as germ tube positive or negative [27, 29]. The only advantage the germ tube test had over HiCrome in our study was with the turn around time of 4 h as against 48 h for HiCrome [27, 28, 30].

Of the two broad species of *Candida* isolated, 74.1% were NAC whilst 25.9% were *C. albicans*. Our observation is similar to those of Okungbowa et al. [46] and Deorukhkar et al. [47], who found that more than 50% of urinary *Candida* isolates belonged to non-*albicans Candida*. In the present study, *C. glabrata* (57.4%), was the most frequently isolated species followed by *C. albicans* (25.9%), *C. krusei* (11.1%) and *C. parapsilosis* (5.6%). As in our study, the predominance of *C. glabrata* over *C. albicans* has been reported in Nigeria and India [46, 48]. However, our result differs from other studies that implicate *C. albicans* as the predominant *Candida* species among pregnant women causing VVC [13, 17, 35]. Further, our results close to those of other studies reporting *C. glabrata* as the second most predominate *Candida* species [13, 35, 49]. From these studies, the presence of *C. glabrata* as the second most predominant *Candida* isolate could explain the gradual increased shift of *C. albicans* to *C. glabrata* in causing VVC. The predominance of *C. glabrata* in VVC in our study could be as a result of self-medication and prolonged antifungal therapy, which may have led to the selection of *C. glabrata* over *C. albicans*, due to their increased resistance to the commonly used antifungal agents that can be purchased over the counter [2, 33].

The current findings showed that 48.1% of *Candida* isolates were resistant to fluconazole by disc diffusion method. A similar resistance pattern was reported in studies from India (19.44%) [35] and from Nigeria (24, 36.2%) [17, 50].

The high resistance pattern of *Candida* isolates to fluconazole in our study may be due to the use of fluconazole mainly for empiric treatment of VVC [20]. As compared to nystatin and voriconazole, all 6 *C. krusei* isolates studied were resistant to fluconazole. This is similar to previous reports that *Candida krusei* is intrinsically resistant to fluconazole [51].

Our study showed that 3.7% isolates were susceptible to nystatin, while 9.3% were resistant with high SDD values. On the contrary, some studies reported that *Candida* species showed no resistance to nystatin, with low SDD values [51–53]. Although fluconazole has been used as the first-line empiric antimycotic drug over the years, its efficacy was very low in the current study very low indicating that there has been a shift of usage from fluconazole to nystatin (Fig. 7). In our study, nystatin was recorded as having the lowest resistance pattern compared to fluconazole and voriconazole. Previous reports suggested that yeasts are universally susceptible to nystatin [54]. However, the low percentage of susceptibility to nystatin in our study can also be attributed to a lack of routine susceptibility testing of nystatin and other antifungal agents.

In this study, all the tested species showed a high susceptibility to voriconazole as compared to fluconazole. *C. krusei* (33.3%) which were resistant to fluconazole, showed high susceptibility to voriconazole. Moreover, a higher frequency of resistance to voriconazole was seen in *C. albicans* than in non-*albicans Candida*, but the difference was not statistically significant. A similar susceptibility pattern was also observed in other studies carried out in India, Turin and Italy [55], and the results of these studies validate the idea that voriconazole could be a promising antifungal agent against active infections caused by *Candida* spp. innately resistant to fluconazole.

## Limitations

Our study has some limitations. First, a limited number of participants were enrolled during the study period due to time constraints. Second, other antifungal disks were not readily available, resulting in the use of only three for this study. Last, due to small groups of participants and information not being readily available in the ANC books, we could not get significant data on patients’ personal hygiene, and could not identify whether the patients were multipara or nullipara.

## Conclusion

Our findings showed high prevalence of VVC among the pregnant women in the study area. We also found *C. glabrata* to be the predominant isolates causing VVC among the pregnant women, followed by *C. albicans*. Most *Candida* isolates in our study were susceptible to voriconazole, which favours its use in empiric treatment considering its broad spectra [56]. However, nystatin recorded the lowest resistance pattern compared to fluconazole and voriconazole. Variations in degrees of susceptibility, SDD and resistance patterns among the three antifungal agents make species identification and antifungal susceptibility testing in routine diagnosis imperative for selection of appropriate antifungal therapy in Ghana. This will prevent treatment failure, thereby avoiding recurring VVC and persistence of resistant strains which can be passed on subpartally to neonates. HiCrome *Candida* differential agar has high efficacy in speciation of *Candida* isolates and should be used in combination with the germ tube test in identification of *Candida* isolates. The outcome of germ tube test, which is rapid, could be given as a preliminary report while awaiting the outcome of HiCrome, which takes about 48 h.

## Data Availability

The dataset generated during and/or analyzed for this current study are not publicly available. However, coded data may be available from the corresponding author on reasonable request.

## Abbreviations

ANC: Antenatal Clinic
BHI: Brain Heart Infusion
*C*: *Candida*
CLSI: Clinical and Laboratory Standard Institute
HMH: Ho Municipal Hospital
HTH: Ho Teaching Hospital
HVS: High Vaginal Swab
MH: Mueller Hinton
mm: Millimeters
OTC: Over The Counter
PID: Pelvic Inflammatory Disease
SDA: Sabouraud Dextrose Agar
SDD: Susceptible-Dose Dependent
Spp: Species
SPSS: Statistical Package for the Social Sciences
μg: Microgram
